# Systematical Analysis of the Protein Targets of Lactoferricin B and Histatin-5 Using Yeast Proteome Microarrays

**DOI:** 10.3390/ijms20174218

**Published:** 2019-08-28

**Authors:** Pramod Shah, Wei-Sheng Wu, Chien-Sheng Chen

**Affiliations:** 1 Graduate Institute of Systems Biology and Bioinformatics, National Central University, Jhongli 32001, Taiwan; 2 Department of Biomedical Science and Engineering, National Central University, Jhongli 32001, Taiwan; 3 Department of Electrical Engineering, National Cheng Kung University, Tainan City 701, Taiwan; 4 Department of Food Safety/Hygiene and Risk Management, College of Medicine, National Cheng Kung University, Tainan City 701, Taiwan

**Keywords:** Lactoferricin B (Lfcin B), Histatin-5, antimicrobial peptides (AMPs), antifungal activity, proteome microarray, synergy

## Abstract

Antimicrobial peptides (AMPs) have potential antifungal activities; however, their intracellular protein targets are poorly reported. Proteome microarray is an effective tool with high-throughput and rapid platform that systematically identifies the protein targets. In this study, we have used yeast proteome microarrays for systematical identification of the yeast protein targets of Lactoferricin B (Lfcin B) and Histatin-5. A total of 140 and 137 protein targets were identified from the triplicate yeast proteome microarray assays for Lfcin B and Histatin-5, respectively. The Gene Ontology (GO) enrichment analysis showed that Lfcin B targeted more enrichment categories than Histatin-5 did in all GO biological processes, molecular functions, and cellular components. This might be one of the reasons that Lfcin B has a lower minimum inhibitory concentration (MIC) than Histatin-5. Moreover, pairwise essential proteins that have lethal effects on yeast were analyzed through synthetic lethality. A total of 11 synthetic lethal pairs were identified within the protein targets of Lfcin B. However, only three synthetic lethal pairs were identified within the protein targets of Histatin-5. The higher number of synthetic lethal pairs identified within the protein targets of Lfcin B might also be the reason for Lfcin B to have lower MIC than Histatin-5. Furthermore, two synthetic lethal pairs were identified between the unique protein targets of Lfcin B and Histatin-5. Both the identified synthetic lethal pairs proteins are part of the Spt-Ada-Gcn5 acetyltransferase (SAGA) protein complex that regulates gene expression via histone modification. Identification of synthetic lethal pairs between Lfcin B and Histatin-5 and their involvement in the same protein complex indicated synergistic combination between Lfcin B and Histatin-5. This hypothesis was experimentally confirmed by growth inhibition assay.

## 1. Introduction

Treatment of cancer and bacterial infection, as well as immunocompromised patients, has often resulted in fungal infection. An evolutionarily close relationship with humans has limited the development of antifungal drugs. Moreover, the development of antifungal resistance has worsened the condition, causing major health threats [1,2]. All these conditions have led to the urgent need for alternative antifungal agents.

Antimicrobial peptides (AMPs) are key components of the innate immune system that protects the host from invading microorganisms: bacteria, fungi, and viruses. AMPs are short peptides (mostly cationic peptides) with a wide range of antimicrobial activities as well as an adjunct role in immunomodulation and wound-healing [3,4]. The broad-spectrum activity, selective targeting to microorganisms, highly sensitive, multiple modes of the mechanism of action and little toxic effect on human cells makes AMPs a potent alternative to conventional antibiotics [5,6]. Antifungal AMPs are reported to target different fungal cell components [7,8], mostly cell wall and cell membrane, causing leakage of ions and ATPs [9]. AMPs also exert intracellular activities and generation of reactive oxygen species (ROS) that lead to apoptosis/necrosis and cell death [10,11]. Despite multiple mechanisms of antifungal AMPs, very few targets have been reported.

Lactoferricin B (Lfcin B) is a 25-residue peptide (derived from bovine lactoferrin; residues 17–41) with a net positive charge of +8. Lfcin B has twisted antiparallel β-sheet structure containing hydrophilic and hydrophobic residues on the alternative strand [12]. Lfcin B is also detected in the human gut as a result of bovine milk consumption [13]. Lfcin B has antimicrobial activity against bacteria, fungi, cancer cells, and others. The minimum inhibitory concentration (MIC) of Lfcin B against *Escherichia coli* (*E. coli*) wild type and *Saccharomyces cerevisiae* is 15.6–31.2 µg/mL and 0.68 µg/mL, respectively [14,15]. Lfcin B has been reported to exert intracellular activity against yeast [14,16]; however, the intracellular binding targets are unknown. Moreover, the hydrophobic residue is crucial for the translocation of Lfcin B through the cell membrane [17]. Proteome microarray is a high-throughput detection platform for the identification of proteome interactions [18]. In this study, we have systematically identified all the yeast-binding proteins of Lfcin B by using yeast proteome microarrays [19,20].

In addition to Lfcin B, we also used yeast proteome microarrays to identify the yeast protein targets of another most potent antifungal AMP, Histatin-5. Histatin-5 is a 24-residue peptide derived from Histatin-3 (family: Histatins) present in human saliva. Histatin-5 has a net charge of +5 and exerts its potential mode of action by targeting intracellular molecules [21,22]. Histatin-5 has no defined structure in water but acquires α-helix structure in dimethyl sulfoxide and aqueous trifluoroethanol [23]. Presence of Histatin-5 in the mouth provides initial defense against pathogens and avoid their entry to the human gut [24]. The MIC reported for Histatin-5 against *Saccharomyces cerevisiae* is 128 µg/mL [25]. Despite its potential role, only a few targets of Histatin-5 have been reported [26,27,28,29,30]. Thus, comprehensive identification of Histatin-5 protein targets is needed.

The identified yeast protein targets of Lfcin B and Histatin-5 were subjected to bioinformatics analysis to identify functional enrichment in gene ontology (GO) and analysis of synthetic lethal pairs. Synthetic lethality is the well-studied pairwise genetic mutation combination that occurs between two non-lethal genes’ mutation, where their simultaneous loss causes cell death while the individual gene deletion has no impact on cell viability [31]. Synthetic lethality approach has been recently applied in chemotherapy for the treatment of cancer [32,33] but not fully exploited in case of pathogenic infections in the lack of systematical identification of synthetic lethal pairs. Synthetic lethality is well studied in yeast and other organisms (fruit fly, worm, mouse, and human). Of the entire genes identified from whole-genome sequence in *Saccharomyces cerevisiae* (~6000 genes) only ~1000 are essential genes (known by single-gene deletion mutant) whereas the other non-essential genes, when disrupted in any two combinations (synthetic lethality), cause over 170,000 synthetic lethal pairs [34]. These observations provide the essentiality of every gene in an organism [35]. The canonical explanation governing the mechanism of synthetic lethality is based on the deletion of two genes (synthetic lethal partner) in three ways: parallel pathway, same pathway and reversible steps in a pathway. First, the deletion of two genes working on the parallel pathways that perform the same essential function is redundant in nature (mutually compensatory) [31,36]. Second, deletion of two genes working on the same pathway can be explained by three possible phenomena: (1) partial redundancy where the loss of one gene causes only the partial degradation that might be tolerable but not the loss of both genes; (2) internal redundancy of two steps within a pathway; and (3) double defect in essential protein complex formed by many proteins [34,37,38]. Third, deletion of two genes that target the reversible forward and backward steps of the non-essential pathway [38]. Furthermore, the synthetic lethality approach was applied to determine the effect of pairwise protein essentiality among the identified protein targets of Lfcin B and Histatin-5.

In our previous studies, we have systematically explored the entire *E. coli* protein targets of Lfcin B by used *E. coli* proteome microarrays [39,40]. To analyze the similarity and differences in the pattern of targets of Lfcin B in different species, i.e., yeast and *E. coli*, we have compared the enrichment results of Lfcin B obtained from the protein targets of yeast and the protein targets of *E. coli*.

## 2. Results

### 2.1. Yeast Proteome Microarrays Assay

**Figure 1:**
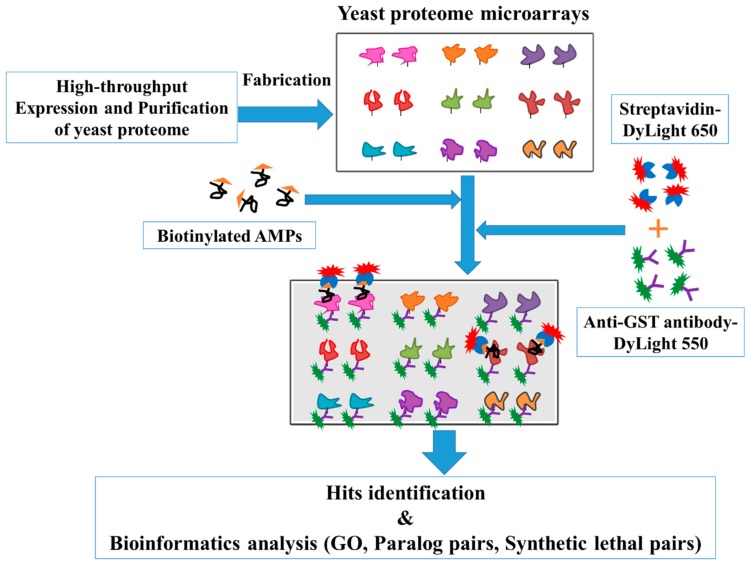
The schematic diagram of this study. Yeast proteome microarrays were fabricated from the ~5800 yeast proteins which were individually expressed and purified from yeast. To identify the protein targets, the fabricated yeast proteome microarrays were probed with biotinylated Lfcin B and Histatin-5, individually. Followed by the probing of DyLight 650 labeled streptavidin and DyLight 550 labeled anti-GST antibody on the yeast proteome microarrays to detect the signal of biotinylated AMPs bound to their specific binding partners and the amount of relative protein on proteome microarray, respectively. The identified hits of Lfcin B and Histatin-5 were systematically analyzed for GO and synthetic lethal pairs by using several online bioinformatics databases (DAVID tools and Synthetic Lethality).

To systematically identify the protein targets of Lfcin B and Histatin-5, high-throughput yeast proteome microarrays were employed. Yeast proteome microarray analysis aids in the parallel identification of protein targets of Lfcin B and Histatin-5 from the entire proteome of yeast. The overall schematic diagram of this study is depicted in Figure 1. Biotinylated Lfcin B and Histstin-5 were individually probed on yeast proteome microarrays and then further probed with DyLight 650-labeled streptavidin and DyLight 550-labeled anti-Glutathione-S-transferase (anti-GST) antibody. DyLight 650-labeled streptavidin was used to detect the biotinylated AMPs that bound to the proteins in yeast proteome microarrays. Because individually purified yeast proteins contain GST tags, anti-GST antibody labeled with DyLight 550 was probed to represent their relative protein amounts on the yeast proteome microarray. Each protein on yeast proteome microarray was printed in duplicate and yeast proteome microarrays assay were conducted in triplicate for Lfcin B and Histatin-5, individually.

**Figure 2:**
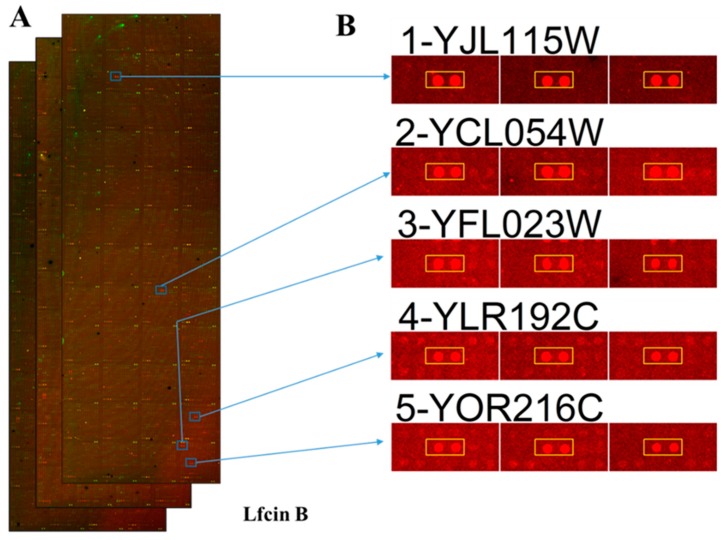

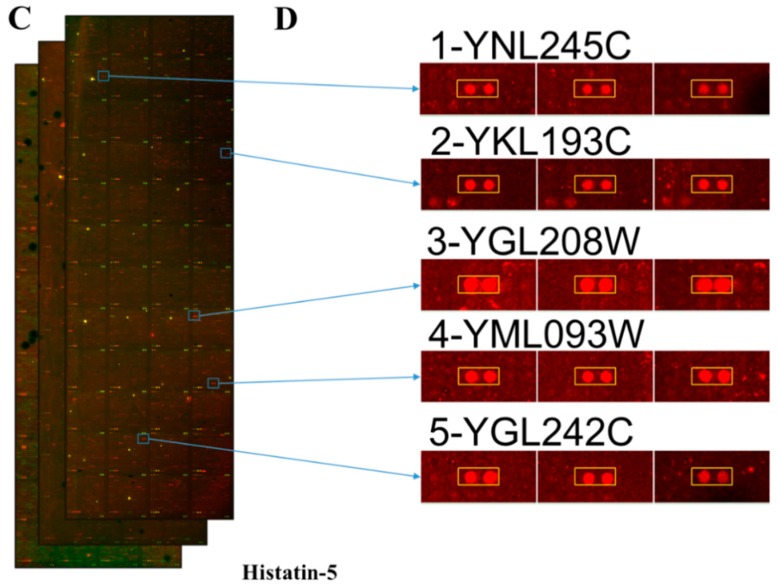
The representative yeast proteome microarray images and the representative hits of Lfcin B and Histatin-5 on yeast proteome microarrays. Images show the microarray and the enlarge hit image of Lfcin B and Histatin-5. Red and green color denotes the signal from DyLight 650 labeled streptavidin and DyLight 550 labeled anti-GST antibody, respectively. Red spots represent the signal of Lfcin B and Histatin-5 on yeast proteome microarrays. (**A**) Triplicate microarray images probed with biotinylated Lfcin B. The position of the representative 5 hits image is shown on the microarray. (**B**) Representative enlarged hits images of Lfcin B. (**C**) Triplicate microarray images probed with biotinylated Histatin-5. (**D**) Representative enlarged hits images of Histatin-5.

**Figure 3:**
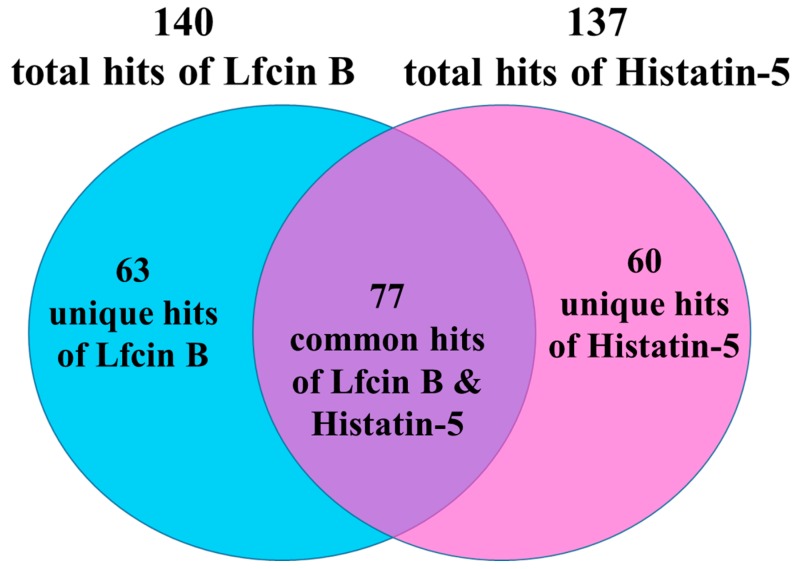
Unique and common hits of Lfcin B and Histatin-5 identified from yeast proteome microarrays. The protein targets of Lfcin B and Histatin-5 identified from yeast proteome microarrays are 140 and 137, respectively. These identified hits of Lfcin B and Histatin-5 were categorized as unique and common hits. Unique protein targets that are present only in Lfcin B are 63 hits whereas the unique protein targets that are present only in Histatin-5 are 60 hits. 77 protein targets were common to both Lfcin B and Histatin-5.

The data obtained from the triplicate yeast proteome microarrays were analyzed together to identify the protein targets of Lfcin B and Histatin-5. After median scaling normalization, hits of Lfcin B and Histatin-5 from yeast proteome microarrays were selected by the local cutoff value that is greater than mean plus two standard deviations and ratio higher than 0.5 of AMP to the anti-GST antibody signal. The total number of protein targets identified for Lfcin B and Histatin-5 from yeast proteome microarrays were 140 and 137, respectively. These hits were all validated by eyeballing their images. The representative images of yeast proteome microarrays assay of Lfcin B (Figure 2A) and the enlarged protein images of representative protein targets of Lfcin B that appeared in the triplicate yeast proteome microarrays assay of Lfcin B are depicted in Figure 2B. Similarly, the representative images of yeast proteome microarrays assay of Histatin-5 on yeast proteome microarrays (Figure 2C) and the enlarged protein images of representative protein targets that appeared in the triplicate microarray assays of Histatin-5 on yeast proteome microarrays are depicted in Figure 2D. The identified protein targets of Lfcin B and Histatin-5 were analyzed for common and unique targets. Common hits are the protein targets present both in Lfcin B and Histatin-5 whereas unique hits are the protein targets present only in Lfcin B or histatin-5. That means the unique protein targets of Lfcin B are only present in Lfcin B and not in Histatin-5. The Venn diagram (Figure 3) shows that Lfcin B and Histatin-5 have common protein targets of 77. The unique protein targets of Lfcin B and Histatin-5 are 63 and 60, respectively. The total unique and common protein targets of Lfcin B and Histatin-5 from yeast proteome microarrays are displayed in Supplementary Table S1.

### 2.2. Enrichment Analysis in GO Biological Process for the Protein Hits of Lfcin B and Histatin-5

**Figure 4:**
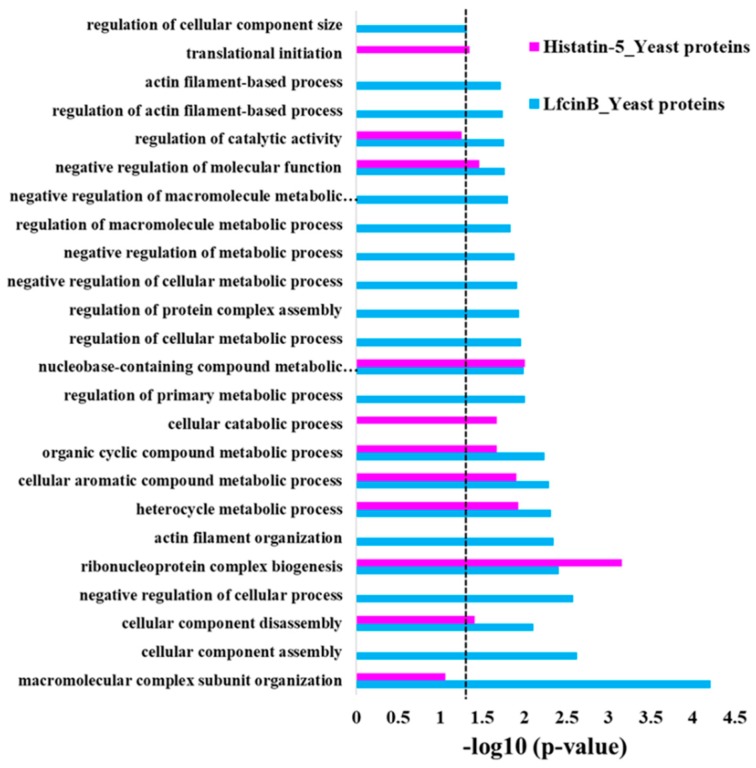
Enrichment in biological process of Lfcin B and Histatin-5 hits obtained from yeast proteome microarrays. The significant enrichment categories in biological process of Lfcin B and Histatin-5 of yeast-binding proteins (dotted line indicates the *p*-value of 0.05).

To know the over-representation proteins with similar function among the protein targets of Lfcin B and Histatin-5, GO enrichment analysis was performed. Using Database for Annotation, Visualization and Integrated Discovery (DAVID) online database [41], we obtained GO enrichment results in biological processes for the protein hits of Lfcin B and Histatin-5. The results display significant over-representation for the protein targets of Lfcin B and Histatin-5 (*p*-value cutoff of 0.05) in several biological processes (Figure 4). Interestingly, Lfcin B showed enrichment in most of the displayed categories; this depicts the involvement of Lfcin B to a broader targets range than Histatin-5. This might be the reason that Lfcin B has a lower MIC than Histatin-5 does. Lfcin B depicted the most obvious enrichment in “macromolecular complex subunit organization” that regulates macromolecule aggregation or disaggregation to form or alter protein complexes. Lfcin B showed enrichment in several unique categories that are present only in Lfcin B and not in Histatin-5. They are several negative regulation processes that stop or reduce several cellular process, such as: “negative regulation of cellular process”, “negative regulation of cellular metabolic process”, “negative regulation of metabolic process”, and “negative regulation of macromolecule metabolic process” as well as regulation of several unique processes that modulates chemical reactions and pathways involved in normal metabolic processes such as “regulation of primary metabolic process”, “regulation of cellular metabolic process”, “regulation of macromolecule metabolic process”, “regulation of catalytic activity”, and “regulation of cellular component size” (Figure 4). These results indicate that Lfcin B targets proteins that manipulate the transcriptional response. Moreover, Lfcin B showed unique enrichment in the organization of cytoskeletal structures comprised of actin filaments, such as “actin filament organization”, “regulation of actin filament-based process”, and “actin filament-based process” as well as “cellular component assembly”. This over-representation of Lfcin B protein targets shows the possible mechanism of Lfcin B entry inside the yeast and hamper their cytoskeleton. On the other hand, the unique over-representation for the protein targets of Histatin-5 was only observed in two biological processes, i.e., “cellular catabolic process” and “translational initiation”. These two categories are related to the breakdown of substances and formation of an initial translational complex of the ribosome, mRNA, respectively.

Common enrichment for the protein targets of Lfcin B and Histatin-5 shows the conserved targets of these two AMPs on yeast. “Ribonucleoprotein complex biogenesis” is the well-conserved protein targets of Lfcin B and Histatin-5 that are involved in RNA-protein complex formation. Several compound metabolic processes were also conserved such as “cellular aromatic compound metabolic process”, “organic cyclic compound metabolic process”, “nucleobase-containing compound metabolic process”, and “heterocycle metabolic process”. “Negative regulation of molecular function” and “cellular component disassembly” is also common enrichment of Lfcin B and Histatin-5 (Figure 4).

### 2.3. Enrichment Analysis in GO Molecular Function and Cellular Component

**Figure 5:**
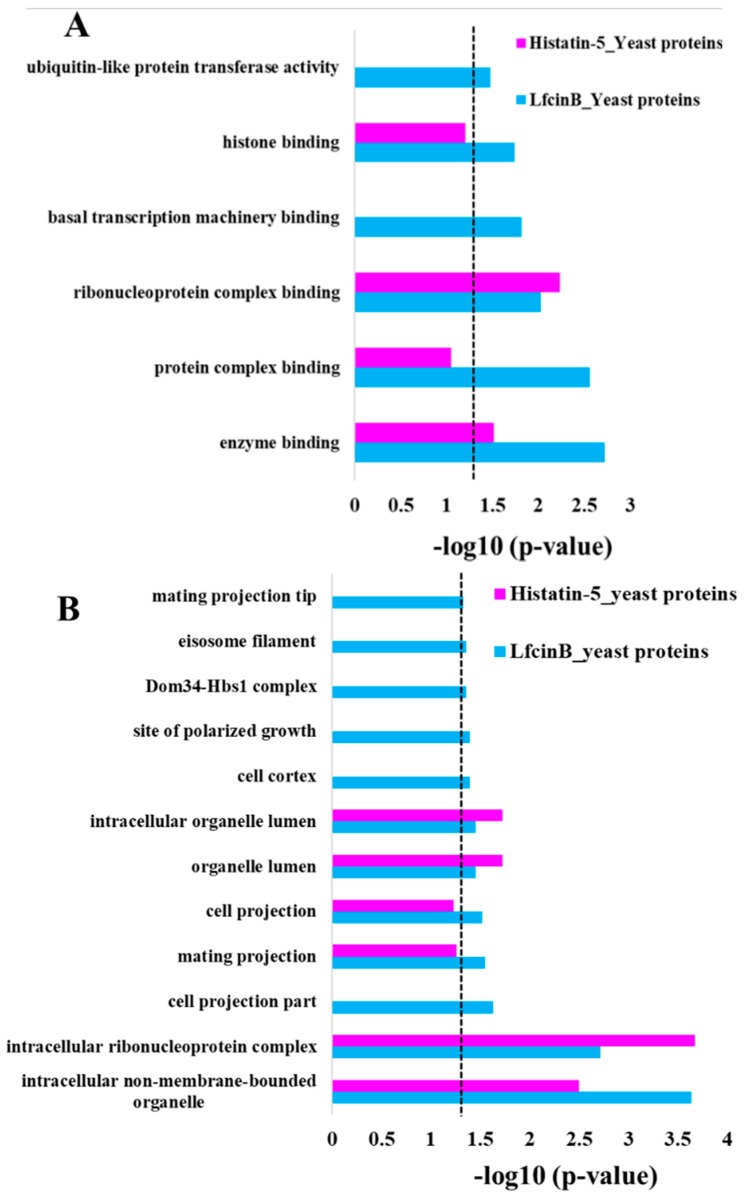
Functional enrichment of Lfcin B and Histatin-5 hits obtained from yeast proteome microarrays. The significant enrichment categories of Lfcin B and Histatin-5 of yeast-binding proteins. (**A**) Enrichment in molecular function (**B**) Enrichment in cellular component (dotted line indicates the *p*-value of 0.05).

In addition to GO enrichment in the biological process, we also analyzed GO enrichment in molecular function and cellular component for the protein targets of Lfcin B and Histatin-5 (Figure 5). The analysis of over-representation for the protein targets of Lfcin B in GO molecular function (Figure 5A) showed unique enrichment in “histone binding” (related to DNA binding protein), “basal transcription machinery binding” (related to proteins of basal transcription factors and RNA polymerase core enzyme) indicating the involvement of Lfcin B in several proteins related to DNA and RNA binding that regulate gene expression in yeast. Protein targets of Lfcin B also showed unique over-representation in “protein complex binding” and “ubiquitin-like protein transferase activity”. On the other hand, Histatin-5 showed no unique enrichment. However, common enrichment for Lfcin B and Histatin-5 were observed in “ribonucleoprotein complex binding” and “enzyme binding”. The enrichment in “ribonucleoprotein complex binding” is similar to the enrichment observed in biological process which indicates the highly conserved targets of Lfcin B and Histatin-5. These results also indicate that the target protein functions of Histatin-5 are also the target protein functions of Lfcin B, and yet Lfcin B targets additional protein functions (Figure 5A).

Figure 5B depicts the GO enrichment in cellular component for the protein targets of Lfcin B and Histatin-5. Unique enrichment of Lfcin B is observed in “cell projection part”, “mating projection”, “mating projection tip”, “cell cortex”, “site of polarized growth”, and “eisosome filament”—these are related to cell and mating projection as well as several signaling pathways which manipulate the development and communication. Lfcin B specifically showed unique enrichment in “Dom34-Hbs1 complex” that indicates its effect on cotranslational mRNA quality control. On the other hand, Histatin-5 showed no unique enrichment. These results also depicted the wider range of over-representation of protein targets in Lfcin B than Histatin-5. In common enrichment, the enriched categories belong to “intracellular non-membrane-bounded organelle”, “intracellular ribonucleoprotein complex”, “organelle lumen”, and “intracellular organelle lumen”. These results showed conserved enrichment of Lfcin B and Histatin-5 in targeting yeast.

### 2.4. Comparison of Lfcin B Protein Targets of Yeast and E. coli

**Figure 6:**
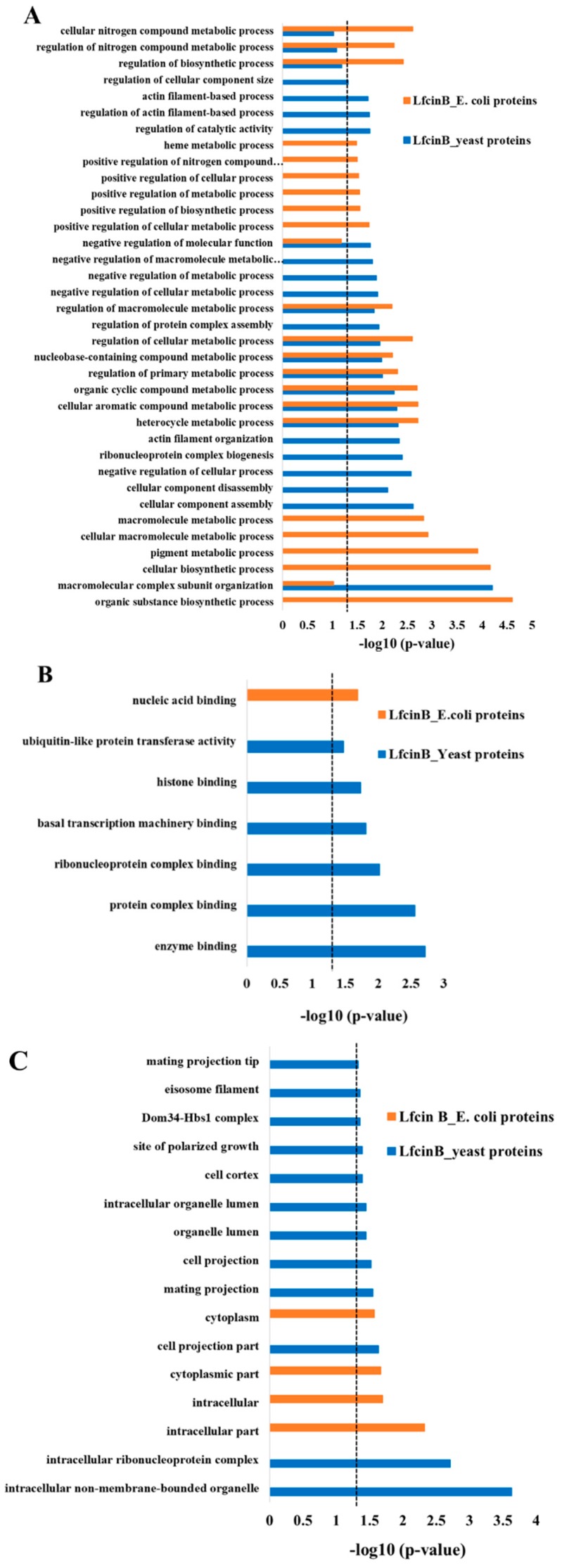
Functional enrichment of Lfcin B hits obtained from *E. coli* proteome microarray and yeast proteome microarrays. The comparison of enrichment categories of Lfcin B target proteins from yeast and *E. coli* proteomes. (**A**) Enrichment in biological process (**B**) Enrichment in molecular function and (**C**) Enrichment in cellular component (dotted line indicates the *p*-value of 0.05).

To analyze the target pattern of Lfcin B in yeast and *E. coli*, we compared the enrichment results of protein targets of Lfcin B obtained from yeast proteome microarrays (in this study) and *E. coli* proteome microarrays (our previous study) [39]. Figure 6 shows the comparison results of GO enrichment in biological process (Figure 6A), molecular function (Figure 6B) and cellular component (Figure 6C) of the protein targets of Lfcin B in yeast and *E. coli*. In biological process, the protein targets of Lfcin B from yeast and *E. coli* showed only 7 common enrichment categories whereas 15 and 14 unique enrichment categories, respectively (Figure 6A). Several unique enrichments result of Lfcin B for yeast and *E. coli* indicated that Lfcin B exerted different target patterns in yeast and *E. coli*. These results also mean that the mechanism by which Lfcin B inhibits yeast is different from *E. coli*. Some targets of Lfcin B are conserved between yeast and *E. coli* and showed common enrichment that were mostly related to metabolic processes, such as: “heterocycle metabolic process”, “cellular aromatic compound metabolic process”, “organic cyclic compound metabolic process”, “regulation of primary metabolic process”, “nucleobase-containing compound metabolic process”, “regulation of cellular metabolic process”, and “regulation of macromolecular metabolic process”.

The difference in the mechanism of actions of Lfcin B in yeast and *E. coli* were also obtained by comparing enrichment results of molecular function and cellular component. The result depicted in Figure 6B shows the enrichment categories in molecular function. Lfcin B showed single unique enrichment in “nucleic acid binding” for the protein targets of *E. coli* whereas for protein targets of Lfcin B from yeast showed significant enrichment in several functions related mostly to protein-binding, such as “enzyme binding”, “protein complex binding”, “ribonucleoprotein complex binding”, “basal transcription machinery binding”, “histone binding” and “ubiquitin-like protein transferase activity”. It is very interesting to observe that Lfcin B targets most yeast proteins with protein-binding function and most *E. coli* proteins with nucleic acid binding function. In case of cellular component (Figure 6C), *E. coli* protein targets of Lfcin B showed enrichment in the cytoplasm and intracellular whereas yeast protein targets of Lfcin B were enriched in several categories related to cell, ribonucleoprotein, organelle lumen, and mating projection, as well as several signaling pathways.

The above enrichment comparison results of protein targets of Lfcin B from yeast and *E. coli* showed several functional enrichments in yeast than that of *E. coli*, indicating the wider targets of Lfcin B in the case of yeast than *E. coli*. Thus, Lfcin B causes higher effect against yeast than that of *E. coli*.

### 2.5. Identification of Synthetic Lethal Pairs Targeted by Lfcin B and Histatin-5

The identification of synthetic lethal pairs is critical for deciphering the mechanism of action as they exert lethal effects on growth. Synthetic lethality database [42] was used to identify the synthetic lethal pair for the protein targets of Lfcin B and Histatin-5. Within the protein targets of Lfcin B, we identified 11 synthetic lethal pairs. All the identified synthetic lethal pairs within the protein targets of Lfcin B are shown in Table 1. Among these 11 synthetic lethal pairs of Lfcin B, one paralog pair (SIS2–VHS3) was also identified. Within the protein targets of Histatin-5, we identified a total of three synthetic lethal pairs. Synthetic lethal pairs cause lethal effect on yeast growth, and the higher the number of identified synthetic lethal pairs, the higher is the lethality impact on growth. A total of 11 synthetic lethal pairs identified within the yeast protein targets of Lfcin B would cause intense lethality effect on yeast than the three synthetic lethal pairs identified within the protein targets of Histatin-5. This might also be the potential reason for the previously observed lower MIC of Lfcin B against *Saccharomyces cerevisiae* than that of Histatin-5.

**Figure 7:**
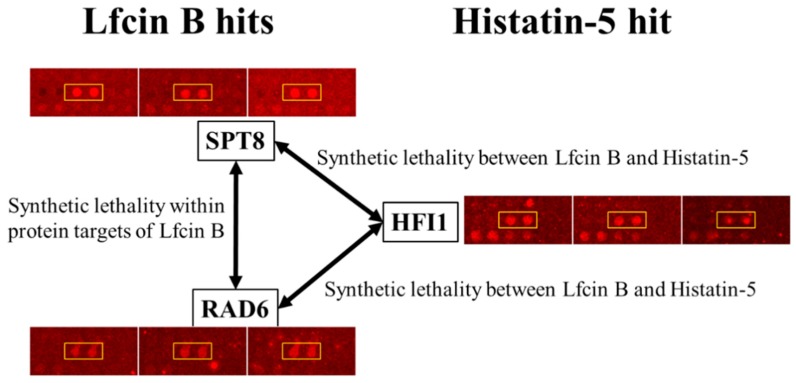
Synthetic lethality and enlarge protein images of SPT8, RAD6, and HFI1 on yeast proteome microarray. The enlarged protein images of two synthetic lethality pairs identified between the unique hits of Lfcin B and Histatin-5 as well as the synthetic lethality pair within the protein targets of Lfcin B.

We further analyzed the synthetic lethal pair between the unique protein targets of Lfcin B and Histatin-5 by using synthetic lethality database. Two synthetic lethal pairs were identified between the unique protein targets of Lfcin B and Histatin-5. The identified synthetic lethal pairs and the function of individual proteins are depicted in Table 2. Also, the individual protein images and their signals from triplicate yeast proteome microarrays were validated by eyeballing and the enlarged protein images are illustrated in Figure 7. The first synthetic lethal pair (SPT8 and HFI1) identified between the unique protein targets of Lfcin B and Histatin-5 are the subunits of the Spt-Ada-Gcn5 acetyltransferase (SAGA) complex. SAGA complex is involved in histone modification that characterizes histone acetyltransferase and histone deubiquitinase [43]. The synthetic lethality caused by Lfcin B and Histatin-5 treatment will hamper the structural integrity as well as the histone modification function of SAGA complex. Moreover, SPT8 and HFI1 subunits of SAGA complex are reported to bind with TATA-binding protein (TBP) and function in delivering TBP to TATA box [44]. Thus, this lethal pair will also effect initiation of transcription processes.

The second synthetic lethal pair (RAD6 and HFI1) identified between Lfcin B and Histatin-5 also targets the histone modification function. RAD6 (ubiquitin associated enzyme E2), together with BRE1 (ubiquitin enzyme E3) is known to modify histone H2B-lysine at position 123 with ubiquitin [45]. This monoubiquitination of histone H2B is not for H2B degradation, rather the ubiquitin in H2B (H2B~Ub) server as a signal for various aspects of gene expression, such as initiation and elongation of transcription as well as DNA replication and repair [46]. Ubiquitination in H2B-lysine 123 is reversed by SAGA complex that functions as deubiquitination of H2B~Ub and causes acetylation of histone [47]. H2B~Ub up-regulate histone H3-lysine 4 methylation and down-regulate histone H3-lysine 36 methylation, whereas SAGA deubiquitination of H2B acts in the opposite way and reduce H3-lysine 4 methylation and increase H3-lysine 36 methylation levels. Together, ubiquitination and deubiquitination are involved in transcriptional activation [48]. Lfcin B targeting RAD6 would hamper histone H2B ubiquitination whereas Histatin-5 targeting HFI1 would disrupt SAGA complex and cause functional defect (deubiquitination and acetylation) of SAGA complex to modify histone H2B. Moreover, SPT8 and RAD6 are both the protein targets of Lfcin B and were also reported to have synthetic lethality (Table 1). The mechanism of synthetic lethality is similar to RAD6 and HFI1 (as explained above).

It is known that synthetic lethal pairs identified during treatment with two drugs may have a synergistic effect [49,50,51], as the two drugs targeting the individual protein of synthetic lethal pair cause extra synthetic lethality. The two synthetic lethal pairs identified between the unique protein targets of Lfcin B and Histatin-5 ensure additional synthetic lethal effects on yeast growth that are not observed with individual treatment of Lfcin B or Histatin-5. Moreover, both these identified synthetic lethal pairs are involved in the structure and function of SAGA protein complex that regulates gene expression by modifying histone. Additional synthetic lethal pairs, as well as the same protein complex target, will cause a significantly higher lethality effect on yeast by the combined treatment of Lfcin B and Histatin-5. Thus, we hypothesized synergistic combination between Lfcin B and Histatin-5 on yeast.

### 2.6. Validation of Synergistic Combination between Lfcin B and Histatin-5

**Figure 8:**
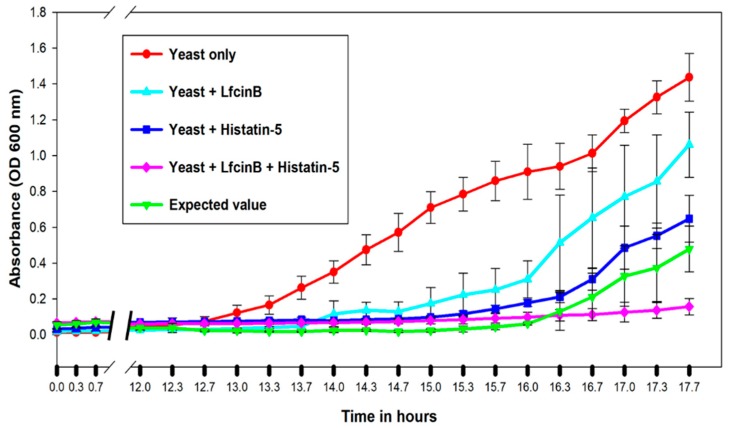
Growth inhibition effect of individual and combination of Lfcin B and Histatin-5 on yeast. Yeast was grown without AMPs and in the presence of individual and combination of Lfcin B (15 µg/mL) and Histatin-5 (20 µg/mL). Significant inhibition in growth was observed with the combination of Lfcin B and Histstin-5. The combinational inhibition of Lfcin B and Histatin was significantly lower than the expected value of Lfcin B and Histatin-5 combination calculated from the individual inhibition of Lfcin B and Histatin-5, concluding the synergy combination between Lfcin B and Histatin-5.

Based on two pairs of synthetic lethality interactions targeted by unique hits of Lfcin B and Histatin-5, we predicted a synergistic effect between Lfcin B and Histatin-5. This prediction was experimentally validated by the growth inhibition curve in the presence of individuals and a combination of Lfcin B and Histatin-5 (Figure 8). A combination of Lfcin B and Histatin-5 clearly showed drastic inhibition effects on yeast growth curves compared with individual AMPs. To show the result obtained is synergistic, we calculated the expected combinational value of Lfcin B and Histatin-5 from the anticipated optical density (OD) values of the inhibitory effect of the individual Lfcin B and Histatin-5 on yeast growth curves in sequence (i.e., OD of yeast without AMP multiplied by the percentage of the remaining yeast after the treatment of Lfcin B and further multiplied by the percentage of the remaining yeast after the treatment of the Histatin-5). A significant difference was observed between the expected combination value and experimental combination value of Lfcin B and Histatin-5. This result demonstrated the synergistic combination of Lfcin B and Histatin-5 against yeast growth.

## 3. Discussion

Lfcin B and Histatin-5 are potent AMPs with antifungal activities [16,52]. Given the lack of entire targets knowledge, the potential mechanisms of antifungal AMPs are not fully explored. In this study, we have systematically identified the entire yeast protein targets of Lfcin B and Histatin-5 by using yeast proteome microarrays. A total of 140 and 137 protein targets of Lfcin B and Histatin-5 were identified from yeast proteome microarrays, respectively. Lfcin B and Histatin-5 can penetrate the yeast cell envelope so we assume that the bioavailability of Lfcin B and Histatin-5 on the yeast proteome microarrays will be similar to yeast in in vivo.

Earlier studies with *Candida albicans* have shown the binding of Histatin-5 to the cell surface proteins SSA1 and SSA2 [53,54]. In our yeast proteome microarray, both SSA1 and SSA2 were absent, thus we did not identify them in our hit list. Histatin-5 is reported to target mitochondria [26,55] and generate and release reactive oxygen species (ROS) that causes cell death [52]. Thus, we looked for the protein targets of Histatin-5 that are involved in mitochondria. We identified seven proteins: AIM21, AIM26, DOA1, YMR26, ETR1, ATP5, and RML2, which are involved in several mitochondrial functions, and six of them (not AIM21) are localized inside the mitochondria. AIM21 is a cytoplasmic protein that helps mitochondrial migration along actin filaments. This result depicted that our finding not only supported the previous finding but also provided the complete targets of Histatin-5 in mitochondria.

Ergosterol is the major fungal membrane sterol essential for fungal cell viability and is absent in humans [56]. Most of the antifungal drugs on the market target ergosterol biosynthesis enzymes [57]. Among the hits of Lfcin B, Lanosterol synthase (ERG7) belonging to ergosterol biosynthesis was identified. Molsidomine, a drug used as a vasodilator for the treatment of angina, was reported to target ERG7 and showed potential antifungal activity [58]. However, no antifungal drug targeting ERG7 is commercially available on the market.

Furthermore, two protein targets belonging to the ergosterol pathway were identified individually in Lfcin B and Histatin-5 by lowering the hit identification cutoff value from 2SD to 1SD. Lfcin B targeted ERG1 and ERG7 whereas Histatin-5 targeted ERG1 and ERG12. These protein targets were individually validated by eyeballing and the enlarged images of these proteins shown in Supplementary Figure S1. ERG1, the common target of Lfcin B and Histatin-5, is the first enzyme in ergosterol biosynthesis and a well-known target of antifungal drugs class, Allylamines (naftifine and terbinafine) [59]. ERG7 is the second enzyme in the ergosterol biosynthesis after ERG1. ERG1, ERG7, and ERG12 are essential genes in the biosynthesis of ergosterol and their individual deletion is lethal for yeast. Deletion of Erg7 results in accumulation of ERG9, which hampers squalene production and amounts to ergosterol biosynthesis. String analysis showed protein–protein interaction between ERG1, ERG7, and ERG12 (data not shown). ERG7 and ERG12 have not been exploited as the target; thus, Lfcin B and Histatin-5 can be a potential target to cope with emerging antifungal drug resistance fungi.

Synthetic lethality approach has recently gained attention for its potential to understand and design medication for cancer [32,33]. Synthetic lethality describes a useful pairwise interaction, where the simultaneous deletion of both the component of the pair cause growth defects but the deletion of an individual component has no effect on growth [31]. We have used a synthetic lethality approach to identify synthetic lethal pairs within the protein targets of Lfcin B and Histain-5. The number of synthetic lethal pairs identified within the protein targets of Lfcin B and Histatin-5 might demonstrate the potential effect it exerts on yeast. Within the protein targets, 11 synthetic lethal pairs in Lfcin B and three synthetic lethal pairs in Histatin-5 were identified. These results showed that Lfcin B exerts a more lethal effect than Histain-5. Interestingly, it is reported that the MIC of Lfcin B is lower than Histatin-5. Thus, our analysis provided the mechanism of lower MIC of Lfcin B against yeast than Histatin-5, based on the number of identified synthetic lethal pairs. Our analysis also identified two synthetic lethal pairs between the unique protein targets of Lfcin B and Histatin-5. Apart from the individual synthetic lethal pairs of Lfcin B and Histatin-5, the combination of Lfcin B and Histatin-5 targeted two additional synthetic lethal pairs. These additional synthetic lethal pairs are the subunits of SAGA protein complex and are also involved in similar functions. The treatment of yeast with Lfcin B and Histatin-5 might cause a greater inhibition effect as they will shut down the structure and function of SAGA complex. Together, Lfcin B and Histatin-5 might exert synergistic combinations. We performed in vivo growth inhibition assays to test our synergistic combination hypothesis between Lfcin B and Histatin-5. The significantly higher inhibition results in the combined treatment of Lfcin B and Histain-5 (experimental data) than the expected combinational value confirmed the synergistic combination between Lfcin B and Histatin-5.

In this study, we have explored the entire biological targets of Lfcin B and Histatin-5 using yeast proteome microarrays. The identified protein hits were analyzed to observe over-representation in different GO enrichment categories. The results showed wider GO enrichment results for Lfcin B than Histatin-5 in all the three categories of the biological process, molecular function, and cellular component. Moreover, 11 synthetic lethal pairs were identified within the protein targets of Lfcin B whereas only three synthetic lethal pairs were identified within the protein targets of Histain-5. Both these results proved the higher lethal effect of Lfcin B on yeast than Histatin-5. This might result in lower MIC of Lfcin B than Histatin-5 which is in accordance with the previously reported results. Two synthetic lethal pairs were identified between the unique protein targets of Lfcin B than Histatin-5. Thus, we hypothesized a synergistic combination between Lfcin B and Histatin-5. Based on the hypothesis, we designed an inhibition assay to test it and successfully validated our hypothesis. In the future, we will further explore the mechanism of actions of other AMPs with antifungal activities.

## 4. Materials and Methods

### 4.1. Expression and Purification of the Entire Yeast Proteome

Yeast entire proteome was expressed and purified using the previously reported protocol [19,20]. Briefly, yeast library consists of ~5800 open reading frames, individually cloned in high-copy URA3 expression vector with Glutathione-S-transferase–poly-histidine (GST–HisX6) tag. These clones use galactose-inducible GAL1 promoter to produce GST-HisX6 fusion proteins. Yeast clones were stored in 96-well format at −80 °C. For protein expression, yeast clones were first grown on SC-URA3-glucose agar plate at 30 °C for 48 h. Colonies were transferred to SC-URA3-glucose medium in 96-deep well plates and incubated at 30 °C for 24 h. Yeast clones were sub-cultured in SC-URA3-raffinose medium in 12 channel reservoirs (a single 96-well plate requires eight 12-channel reservoir plates; in total, the yeast library contains 66 plates of 96 well plates) and incubated at 30 °C for 14–16 h (till Optical Density at 600 nm reached to 0.6–1.0). For protein expression, 2% galactose was added to the cultures in SC-URA3-raffinose medium and further incubated for 4 h. Cells in 12-channel reservoirs were centrifuged at 4000 rpm for 2 min and cells were re-suspended in 800 µL cold water. Each strain in 12-channel reservoir was pooled together in each well of 96-deep well plates. Cells were harvested by centrifuging at 4000 rpm for 2 min and stored at −80 °C ahead of protein purification.

For protein purification, yeast colonies were ruptured, and GST tag proteins were purified by using glutathione (GSH) beads, following standard protocol. Briefly, 200 µL of 0.7 mm zirconia beads (Biospec Products, Inc., Bartles- ville, OK, USA) were loaded in each well of 96-deep well plate with cell pellets. Freshly prepared 400 µL of lysis buffer with protease inhibitors (1 mM phenylmethylsulfonyl fluoride (PMSF), 50 µM calpain Inhibitor I (LLnL), 1 µM MG132 and 50 × dilution of Roche protease inhibitor (Roche Molecular Biochemicals, Basel, Switzerland)) were added in each well and cell were thaw at 4 °C for 15 min. If not stated otherwise, the chemicals used here were purchased from Sigma-Aldrich (Saint Louis, MO, USA) Again, 400 µL of freshly prepared lysis buffer with protease inhibitors was added and the 96-deep well plates were vortexed at 4 °C for 30 min. After centrifugation at 4000 rpm for 15 min, the supernatant was transferred to new 96-deep well plates. Pre-washed 100 µL of GSH Sepharose 4B beads (GE Healthcare, Chicago, IL, USA) was added to each well and plates were sealed tight using 96-well cap mats (Thermo Fisher Scientific, Waltham, MA, USA). The plates were placed vertically on the shaker and shaken gently (80 rpm) at 4 °C for 80 min. Homogeneous mixtures in 96-deep well plates were transferred to 96-well filter plates (Thermo Fisher Scientific, Waltham, MA, USA) with a filter pore size of 20 µm. The contents were washed with wash buffer I and II and gentle spin dry (1000 rpm for 1 min) to remove extra wash buffer. The bottoms of filter plates were sealed and 50 µL of elution buffer containing reduced GSH was added in each well. 96-well filter plates were shaken vigorously at 4 °C for 1 h. The elutes (proteins) were collected in 96-well receiver plates by centrifugation at 4000 rpm for 2 min. To determine the purity and concentration of purified proteins, proteins were randomly selected for SDS-PAGE and Coomassie blue staining was used afterward.

### 4.2. Fabrication of Yeast Proteome Microarrays

Previously reported protocol was applied to fabricated yeast proteome microarrays [20]. Briefly, the entire purified yeast proteins in 96-well format were transferred to 384-well format by using Liquidator 96 manual pipetting system (Mettler-Toledo Rainin, LLC Oakland, CA, USA). Before printing, the optimal concentrations of landmarks assisted to align blocks on the microarray were determined and tested. In a cold room, the individual proteins and landmarks were printed in duplicate on aldehyde-coated glass slides by using CapitalBio SmartArrayer™ 136 (CapitalBio Corporation, Beijing, China). CapitalBio SmartArrayer is a high-throughput microarray spotter with 48 pins and print 48 proteins at the same time. After printing, the chips were left in the cold room for overnight and finally stored at −80 °C. To monitor the shape, size, and uniformity for each protein spot on a chip, DyLight 550 conjugated anti-GST monoclonal antibody (Rockland Immunochemicals, Gilbertsville, PA, USA) was probed, washed and scanned with LuxScan^TM^ (10K Microarray Scanner; CapitalBio Corporation, Beijing, China).

### 4.3. Yeast Proteome Chip Assays with Lfcin B and Histatin-5

N-terminal biotin labeled (biotinylated) AMPs (Lfcin B and Histatin-5) were purchased from Kelowna International Scientific Inc. (Taipei, Taiwan). AMPs were aliquot and stored at −80 °C. Below are the amino acid sequences of Lfcin B and Histatin-5 used in this study.

Lfcin B (25 residues): H2N–FKCRRWQWRMKKLGAPSITCVRRAF–COOH
Histatin-5 (24 residues): H2N–DSHAKRHHGYKRKFHEKHHSHRGY–COOH

Yeast proteome microarray was first blocked with 3% bovine serum albumin (BSA; Sigma-Aldrich, Saint Louis, MO, USA) in 1X PBS for 1 h at room temperature (RT) with shaking (50 rpm). Chips were washed once with 300 mL PBS-T (0.05% Tween 20) at RT with shaking (50 rpm) for 5 min. Biotinylated AMP (5 µM) was diluted in 1% BSA in 1X PBS and probed individually on yeast proteome microarray with incubation for 1 h at RT with shaking (50 rpm). Chips were washed once with 300 mL PBS-T at RT with shaking (50 rpm) for 5 min. To detect signal from GST tag in yeast proteins and biotinylated AMP bound to yeast proteins, DyLight™ 550 labeled anti-GST antibody (Abcam, Cambridge, UK) and DyLight™ 650 labeled streptavidin (Thermo Fisher Scientific, Waltham, MA, USA) were probed on yeast proteome microarray and incubated with shaking (50 rpm) at RT for 1 h. Finally, the chips were washed for 3 times with 300 mL TBS-T at RT with shaking (50 rpm) for 5 min each. The chips were dried using centrifuge at 1000 rpm for 1 min and scanned with LuxScan.

The chip scan files (TIF format) were opened with GenePix Pro 6.0 software (Axon Instruments, Foster City, CA, USA) and each protein spots on yeast proteome microarray were aligned with their protein names. Binding signals of protein spots on yeast proteome chips were exported in GPR files and later opened with excel files to analyze the results. Median scaling normalization was applied to normalize the signals of Lfcin B, Histatin-5, and anti-GST antibody. After the normalization, the relative binding ability of each AMP to each protein was estimated by the ratio of the fluorescence intensity of each AMP to the anti-GST antibody. The hits were defined as positive only if they met two cutoffs below. First, the signal is higher than the local cutoff, which was defined as one standard deviation (SD) above the signal mean for each spot. Second, the fold change of each AMP signal to anti-GST antibody should be higher than 0.5. Thus, the protein hit list was generated according to the relative binding ability.

### 4.4. Bioinformatics Analysis of Gene Ontology

The *Saccharomyces* Genome Database (SGD) (https://www.yeastgenome.org/) and Universal Protein Resource (UniProt) database (https://www.uniprot.org/) provide comprehensive biological information on particular species [60,61]. SGD provides genome-wide information only on *Saccharomyces cerevisiae* whereas UniProt covers a large group of organisms from prokaryotes to eukaryotic, including *Saccharomyces cerevisiae*. These databases were used for the identification of recommended name (Standard name) from ordered locus names (Systematic name) as well as a brief description of proteins and related function and location inside the cell.

The DAVID (https://david.ncifcrf.gov/) [41] is an online database that provides several tools such as GO, Pfam domain analysis and other enrichment analysis. DAVID was used for comprehensive analysis of functional annotations of positive hits of Lfcin B and Histatin-5, individually. This investigation was necessary to understand the biological meaning of the identified yeast proteins targets of Lfcin B and Histatin-5. Here, we used the GO terms to identify biological processes, cellular component and molecular function, as well as the statistical analysis, was performed via *p*-value to determine the significant enrichment in each category.

### 4.5. Bioinformatics Analysis of Synthetic Lethality Pairs

Synthetic Lethality database (http://histone.sce.ntu.edu.sg/SynLethDB/index.php) (SynLethDB) is the open-source database of synthetic lethality gene pairs from different source organisms [42]. SynLethDB was used for the comprehensive identification of synthetic lethality pairs between the protein hits of Lfcin B and Histatin-5.

### 4.6. Growth Inhibition Assay on Yeast

Yeast (*Saccharomyces cerevisiae* Y258) was incubated with or without AMPs to observe individual and combination inhibition effect of AMP. Yeast was grown on yeast extract peptone dextrose (YPD) agar plate for 48 h at 30 °C. A single colony was transferred into YPD liquid media and incubated with shaking for 24 h at 30 °C. Optical density at 600 nm (OD_600_ nm) was detected for culture grown for 24 h and the culture was diluted to approximately 0.001 in YPD medium. The diluted culture of yeast was added in Nunc^TM^ F 96 well plate (Nalge Nunc International, Rochester, NY, USA) containing an individual and combined number of AMPs in specific wells. The working concentration of Lfcin B and Histatin-5 were 15 µg/mL and 20 µg/mL, respectively. The 96-well plate was incubated at 30 °C in an automated Synergy 2 Multi-Mode Microplate Reader (BioTek Instruments Inc., winooski, VT, USA) and the growth was monitored on a regular interval of 20 min (Shaking for 15 s prior to reading) at OD_600_ nm. Data were collected automatically using Gen5™ reader control and data analysis software (BioTek Instruments Inc., winooski, VT, USA). To show graphic representation of growth inhibition assay, the data were plotted using Sigmaplot. Expected value at given interval (E_XY_) = OD_0_ *(OD_X_/OD_0_) *(OD_Y_/OD_0_) [62]. Where OD_0_ is the optical density of yeast in absence of AMP, OD_X_ is the optical density of yeast in the presence of Lfcin B and OD_Y_ is the optical density of yeast in the presence of Histatin-5 at a given interval of time.

## Figures and Tables

**Table 1:** Synthetic lethal pairs within the total protein targets of Lfcin B and Histatin-5. * denotes paralog pairs among the identified synthetic lethal pairs.

Synthetic Lethal Pairs within the Total Protein Targets of Lfcin B	Synthetic Lethal Pairs within the Total Protein Targets of Histatin-5
ASF1–RAD50	ASF1–RAD50
SIS2–VHS3 *	ASF1–SPT16
ASF1–ORC2	CDC53–DBP10
RAD6–SPT8	
CDC34–CDC53	
ASF1–SPT16	
RAD50–SLX8	
NPL3–RAD6	
CDC53–DBP10	
CDC34–RAD30	
ABZ1–CDC34	

**Table 2:** Synthetic lethal pairs between the unique hits of Lfcin B and Histatin-5 along with the functions of individual proteins involved in these synthetic lethal pairs.

**Synthetic Lethal Pairs between the Unique Protein Targets of Lfcin B and Histatin-5**	
**SPT8–HFI1**	
RAD6–HFI1	
**Protein target of Lfcin B**	
SPT8	Subunit of SAGA complex; SPT8 bind to TATA box binding protein (TBP) and direct TBP to bind TATA box. TBP binding transcription coregulator involved in histone acetylation, chromatin organization, and both positive and negative regulation of transcription by RNA polymerase II
RAD6	The ubiquitin-conjugating enzyme (E2), involved in ubiquitination of histones and substrates of the N-end rule pathway
**Protein target of Histatin-5**	
HFI1	Subunit of SAGA complex; required for structural integrity; a histone acetyltransferase-coactivator complex that is involved in global regulation of gene expression through acetylation and transcription functions

## Supplementary Materials

The following are available online at https://www.mdpi.com/1422-0067/20/17/4218/s1, Figure S1: Image of hits belonging to Ergosterol biosynthesis; Lfcin B (ERG7 & ERG1) and Histatin-5 (ERG 12 & ERG1). Table S1: Two standard deviation (2SD) hits of Lfcin B and Histatin-5 from yeast proteome microarrays.
