# Supplementary material for: Systematical Analysis of the Protein Targets of Lactoferricin B and Histatin-5 Using Yeast Proteome Microarrays
Source: Int J Mol Sci. 2019 Aug 28;20(17):4218. doi: 10.3390/ijms20174218 (PMC6747642; doi:10.3390/ijms20174218)
Supplement: Supplementary file 1 [file ijms-20-04218-s001.pdf]

Supplementary Table S1. Two standard deviation (2SD) hits of Lfcin B and Histatin-5 from yeast proteome microarrays.

**Lfcin B _unique hits**		**Histatin-5 _unique hits**		**Common hits of Lfcin B & Histatin-5**		
YER030W	YNL001W	YDR346C	YIL070C	YJL115W	YKL073W	YKL177W
YPL190C	YER116C	YKL193C	YOR238W	YCL054W	YKL081W	YHL007C
YGR086C	YIL128W	YFL034W	YER047C	YFL023W	YLR257W	YDR365W-A
YCR088W	YOR168W	YKL037W	YDR298C	YLR192C	YNL245C	YGL105W
YJR072C	YPL217C	YDL223C	YDR437W	YOR216C	YLR330W	YDR432W
YML062C	YDR270W	YNL303W	YKL213C	YMR235C	YGR076C	YMR140W
YER036C	YJL170C	YKL152C	YBL005W-A	YGL207W	YHR049W	YER087C-A
YLR055C	YHR114W	YNL292W	YJL001W	YOR239W	YNL175C	YIL107C
YNL079C	YCL030C	YFR055W	YGR097W	YML093W	YOR042W	YOR083W
YLR114C	YHR182W	YCR016W	YBL095W	YKL160W	YBR121C	YIL004C
YIL135C	YKR028W	YER048C	YJL118W	YOR054C	YGR126W	YJL051W
YDR054C	YDR152W	YLR410W	YNL217W	YDR496C	YKL091C	YDL129W
YGL058W	YBR296C	YMR133W	YHR088W	YDL153C	YDL070W	YOL070C
YJR141W	YPL098C	YPL254W	YGR067C	YER091C	YBR155W	
YGR187C	YDR168W	YBL036C	YOL005C	YIR003W	YGL256W	
YDR419W	YFR015C	YFR038W	YDR486C	YLL008W	YLR435W	
YKL181W	YAL003W	YDR199W	YOR173W	YGL242C	YDR266C	
YBR060C	YJL028W	YDR353W	YPR152C	YIR012W	YGR080W	
YMR091C	YGR202C	YDR299W	YKL005C	YDL031W	YEL050C	
YKR072C	YKL013C	YER077C	YHL009C	YGL090W	YBR118W	
YHR072W	YBR092C	YEL022W	YIR043C	YMR260C	YHR156C	
YOL054W	YMR173W	YBL055C	YDR442W	YLR249W	YDR312W	
YNL186W	YBR162C	YDL118W	YCR059C	YKR084C	YBR026C	
YPL004C	YLR030W	YDR314C	YDL173W	YBL011W	YER151C	
YNL246W	YMR252C	YDR273W	YLR355C	YNL250W	YLR150W	
YDR362C	YPL258C	YHR172W	YDR413C	YER122C	YBR222C	
YGR167W	YEL025C	YKL162C-A	YKL158W	YDL078C	YDR210W	
YIL104C	YBR272C	YDR047W	YGR059W	YEL012W	YDL132W	
YDR188W	YMR196W	YFL015C		YDL099W	YER180C	
YKL028W	YNR033W	YNL042W		YGR159C	YMR239C	
YML054C	YIL154C	YJL181W		YNL207W	YML007W	
YMR025W		YGR095C		YGL208W	YDR292C	

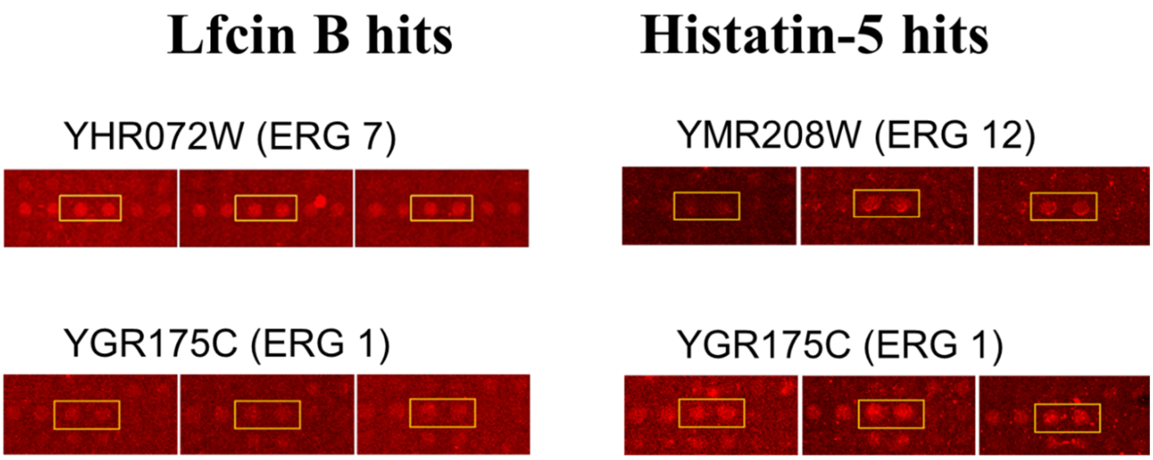

Supplementary Fig. S1. Image of hits belonging to Ergosterol biosynthesis; Lfcin B (ERG7 & ERG1) and Histatin-5 (ERG 12 & ERG1).
